# Spontaneously Occurring Small-Colony Variants of *Staphylococcus aureus* Show Enhanced Clearance by THP-1 Macrophages

**DOI:** 10.3389/fmicb.2020.01300

**Published:** 2020-06-12

**Authors:** Simon M. Stoneham, Daire M. Cantillon, Simon J. Waddell, Martin J. Llewelyn

**Affiliations:** ^1^Department of Microbiology and Infection, Royal Sussex County Hospital, Brighton, United Kingdom; ^2^Department of Global Health and Infection, Brighton and Sussex Medical School, University of Sussex, Brighton, United Kingdom

**Keywords:** *Staphylococcus aureus*, SCV, macrophage, intracellular, phagocyte

## Abstract

*Staphylococcus aureus* is a common cause of chronic and relapsing infection, especially when the ability of the immune system to sterilize a focus of infection is compromised (e.g., because of a foreign body or in the cystic fibrosis lung). Chronic infections are associated with slow-growing colony phenotypes of *S. aureus* on solid media termed small-colony variants (SCVs). Stable SCVs show characteristic mutations in the electron transport chain that convey resistance to antibiotics, particularly aminoglycosides. This can be used to identify SCVs from within mixed-colony phenotype populations of *S. aureus*. More recently, populations of SCVs that rapidly revert to a “wild-type” (WT) colony phenotype, in the absence of selection pressure, have also been described. In laboratory studies, SCVs accumulate through prolonged infection of non-professional phagocytes and may represent an adaptation to the intracellular environment. However, data from phagocytic cells are lacking. In this study, we mapped SCV and WT colony populations in axenic growth of multiple well-characterized methicillin-sensitive and methicillin-resistant *S. aureus* strains. We identified SCVs populations on solid media both in the presence and absence of gentamicin. We generated stable SCVs from Newman strain *S. aureus*, and infected human macrophages with WT *S. aureus* (Newman, 8325-4) and their SCV counterparts (SCV3, I10) to examine intracellular formation and survival of SCVs. We show that SCVs arise spontaneously during axenic growth, and that the ratio of SCV:WT morphology differs between strains. Exposure to the intracellular environment of human macrophages did not increase formation of SCVs over 5 days and macrophages were able to clear stable SCV bacteria more effectively than their WT counterparts.

## Introduction

*Staphylococcus aureus* disease includes common acute skin and soft tissue infections, acute toxin-mediated processes (food poisoning, toxic shock), acute invasive infection such as bacteremia and chronic infections, e.g., of bone and prosthetic joints (Lowy, 1998). Despite rapid treatment with effective antibiotics bacteremia is associated with high rates of metastatic infection and death, and chronic infection is associated with high rates of relapse (Kaasch et al., 2014). Slow-growing phenotypes of *S. aureus*, so-called small-colony variants (SCVs), can be isolated from chronic infections (Proctor et al., 2014). SCVs have been hypothesized to represent a population of persisters that are less immunogenic and more tolerant to antibiotic therapy, and thus may contribute to treatment failure (Tuchscherr et al., 2010; Proctor et al., 2014).

Small-colony variants form small colonies on solid media [<1/10th the area of wild-type (WT) colonies], show reduced pigmentation, and cause reduced haemolysis on blood agar compared to their WT counterparts (von Eiff, 2008). The SCV phenotype can either be stable over successive generations, or rapidly revert to WT colony phenotype. Stable SCVs have been isolated from patients with chronic infections, notably osteomyelitis and cystic fibrosis (Proctor et al., 1995; Besier et al., 2007), with characteristic mutations in components affecting the electron transport chain (Besier et al., 2007; Proctor et al., 2014). There is increasing evidence that non-stable SCVs arise spontaneously at low (c. 1:10,000) frequencies during axenic growth of *S. aureus* (Vulin et al., 2018). Furthermore, it is possible to increase the proportion of the population exhibiting a non-stable SCV phenotype through exposure to antibiotics, particularly aminoglycosides (Edwards, 2012; Vestergaard et al., 2016) and trimethoprim (Kahl et al., 1998), and to conditions mimicking the intracellular environment, including oxidative stress and low pH (Tuchscherr et al., 2011; Leimer et al., 2016). Indeed, several studies have examined intracellular persistence of SCVs within non-professional phagocytes: endothelial cells (von Eiff et al., 1997; Rollin et al., 2017), epithelial cells (Tuchscherr et al., 2011; Leimer et al., 2016), and osteoblasts (Kalinka et al., 2014). This has led to the hypothesis that SCVs represent an adapted intracellular phenotype.

Although *S. aureus* has traditionally been considered to be an extracellular pathogen, the idea that *S. aureus* can survive intracellularly is not new; there is an increasing body of evidence that *S. aureus* can survive within professional phagocytes, notably macrophages (Kubica et al., 2008; Thwaites and Gant, 2011; Jubrail et al., 2016). Macrophages are key effectors of the innate immune system; they are often the first immune cells to encounter bacteria and are therefore likely to play an important role in the initial recognition of, and response to, infection (Rehm et al., 1980). An intracellular lifestyle may protect bacteria from eradication by the immune system, reduce exposure to antibiotics, and underlie dissemination of infection (Thwaites and Gant, 2011). Recently it has been demonstrated that infection of mice with leukocytes containing *S. aureus* led to disseminated infection despite concurrent treatment with antibiotics (Lehar et al., 2015). However, the significance of SCV formation within these cells is unknown.

In the present study, we examined the dynamics of phenotype switching between WT colonies and SCVs *in vitro*. Extended axenic culture gave rise to distinct SCV populations on media with and without gentamicin. We used growth in the presence of gentamicin to identify a stable SCV (SCV3) from the parent strain Newman allowing us to examine the growth kinetics and antibiotic resistance pattern of SCV compared to WT *S. aureus*. To test the hypothesis that SCVs are adapted for intracellular survival, we adapted an *in vitro* infection model using differentiated human THP-1 macrophages. In keeping with previous studies (Jubrail et al., 2016), *S. aureus* was able to persist within macrophages for 5 days. We used phenotypic identification and growth in the presence of gentamicin to identify SCVs arising from intracellular infection. We found no evidence for enrichment of the SCV population during macrophage infection. In addition, macrophages infected with SCV3 showed lower intracellular numbers and more rapid clearance of intracellular bacteria than those infected with the parent strain Newman. Finally, to confirm that this phenomenon was not restricted to Newman strain *S. aureus*, intracellular infections were repeated with *S. aureus* 8325-4 and I10, a SCV constructed through knockout of *HemB* (von Eiff et al., 1997). This study represents the first examination of SCV selection and survival within macrophages.

## Materials and Methods

### Bacterial Strains and Culture

*Staphylococcus aureus* Newman was obtained from the PHE culture collection. MRSA252, NCTC-8532, -12973, and -12493 were obtained from the microbiology department at the Royal Sussex County Hospital. Strains 8325-4 and I10 were donated by Dr. Iain Allen at the University of Brighton. All bacteria, with the exception of I10, were grown to OD_600 nm_ 0.6 in 30 mL Mueller–Hinton Broth (MHB) (Sigma–Aldrich) in 250 mL vented flasks on a shaking incubator at 185 r/min at 37°C. I10 was grown in Tryptic Soy Broth (Sigma–Aldrich) containing 2.5 μg/mL erythromycin (Fisher) to maintain the Erm insertion. I10 was generated as described previously by homologous recombination of an erythromycin resistance cassette into the *HemB* gene resulting in a stable SCV phenotype and has been shown to persist intracellularly in bovine aortic endothelial cells (von Eiff et al., 1997). Extended *in vitro* culture was performed in 250 mL vented flasks on a shaking incubator in 50 mL MHB at 37°C. Growth curves were conducted in 30 mL MHB or RPMI 1640 (Fisher Scientific) supplemented with 10% heat-inactivated fetal calf serum (HIFCS) (Pan Biotech) and 2 mM L-glutamine (Fisher Scientific). OD_600 nm_ was measured using a Jenway 6305 Spectrophotometer in comparison to media only controls.

### Enumeration and Identification of SCVs

Small-colony variants were identified by their appearance on solid media in either the presence or absence of gentamicin. SCVs were identified on antibiotic-free media [Luria-Bertani agar (LA)] where SCV numbers were greater than or similar to the number of WT colonies. In certain experiments, SCVs were also identified on Mueller Hinton agar containing 2 μg/mL gentamicin (MHA+G) as described in Edwards (2012), to allow for enumeration of gentamicin resistant SCVs where WT colony numbers greatly exceeded SCV number. SCVs identified this way were phenotypically similar to SCVs described previously (Proctor et al., 2006). Following identification of SCVs on MHA+G, stability of mutants was determined by serial passage on LA containing no antibiotics. Newman SCV3 was selected as it was able to maintain its SCV phenotype through >8 passages on antibiotic-free media.

### Determination of Minimum Inhibitory Concentrations

MICs were determined by microbroth dilution in MHB. Bacteria were grown in MHB on a shaking incubator at 185 r/min until they reached exponential phase ∼OD_600 nm_ 0.6. Bacteria were diluted to ∼5 × 10^5^ cfu/mL then added to pre-prepared 96-well plates containing either gentamicin, erythromycin, flucloxacillin, rifampicin, or ofloxacin (Sigma); 96 well plates were prepared by twofold serial dilution of antibiotics immediately prior to the experiment. Each experiment was performed three times in triplicate wells. Bacterial growth was determined by measurement of OD_600 nm_ on a Biotek synergy HT plate-reader at either 24 h for WT Newman or 48 h for SCV3. The MIC was defined as the minimum concentration of antibiotic required to completely inhibit growth.

### Cell Culture and Differentiation

The human monocytic leukemia cell line THP-1 was maintained in RPMI 1640 supplemented with HIFCS and 2 mM L-glutamine. Cells were split every 3–4 days to maintain a density of between 3 × 10^5^ and 1 × 10^6^ cells/mL. THP-1 cells were differentiated using 100 nM phorbol 12-myristate 13-acetate (PMA) for 72 h and plated at a density of 2 × 10^5^ cells/mL in 24 well plates to produce a terminally differentiated macrophage phenotype, competent for intracellular killing (Daigneault et al., 2010). On day 3, cells were washed once in supplemented RPMI-1640 and allowed to rest for 5 days before infection.

### Macrophage Challenge With *S. aureus*

Differentiated macrophages were challenged with different strains of *S. aureus* at a multiplicity of infection (MOI) of 1. This method was modified from Jubrail et al. (2016). In brief, frozen stocks of *S. aureus* were thawed, centrifuged at 13,000 r/min for 3 min and resuspended in supplemented RPMI-1640 before 0.5 mL of bacterial suspension or fresh media was added to experimental wells. Cells were allowed to phagocytose bacteria for 3 h at 37°C in 20% CO_2_. After 3 h extracellular bacteria were removed by washing twice in warm supplemented RPMI-1640. Any remaining extracellular bacteria were killed by the addition of 20 μg/mL lysostaphin (Sigma) for 30 min. Lysostaphin was used for elimination of extracellular bacteria as it is not known as a potent inducer of SCVs and does not enter mammalian cells (Kim et al., 2019). Killing of extracellular bacteria was confirmed by control wells containing bacterial suspension but no cells. After 30 min, cells were either lyzed or media was replaced with RPMI containing 2 μg/mL lysostaphin. At specified time points, cells were washed twice in PBS before lysis with 250 μL 2% saponin (Sigma). After 12 min at 37°C, 750 μL PBS was added to wells containing saponin and mixed by vigorous pipetting; 100 μL lysates were serially diluted and plated onto LA overnight or MHA+G for 48–72 h at 37°C before counting colony forming units. Macrophage experiments were performed in triplicate.

### Statistical Analysis

All graphs are represented as mean ± SD or SEM as specified in the figure legend. Statistical analysis was performed using Prism 8 (GraphPad Software, San Diego, CA, United States). Statistical significance was determined as *P* < 0.05. All statistical tests used are listed in the figure legends.

## Results

### SCVs Occur Spontaneously During Axenic Culture of *S. aureus*

**FIGURE 1:**
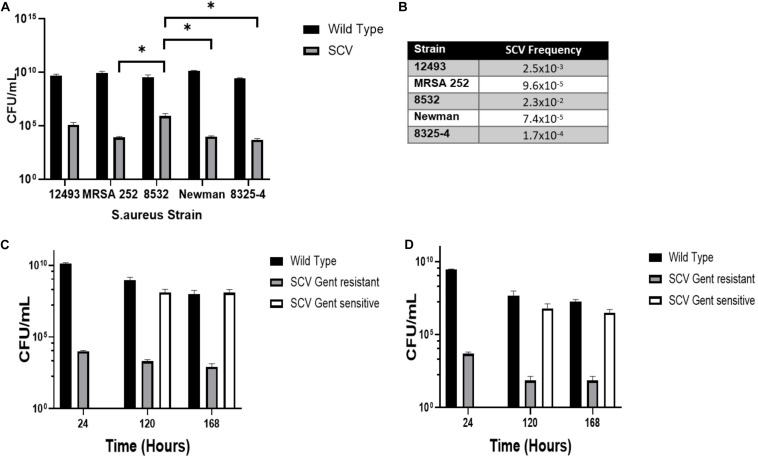
SCVs occur spontaneously during axenic growth of *S. aureus*. **(A)** Multiple strains of *S. aureus* were grown in Mueller Hinton Broth (MHB) for 24 h and CFUs enumerated on media with (MHA+G) and without (LA) gentamicin to select SCV or wild-type colony morphologies, respectively. **(B)** The mean frequency of SCVs to wild-type colonies. **(C)** Newman and **(D)** 8325-4 *S. aureus* strains were cultured in MHB for 7 days and CFUs enumerated on media with (MHA+G) and without gentamicin (LA). After 120 h, a SCV population arose on media without gentamicin. Plots represent the mean of three independent cultures and error bars represent the standard deviations of the mean. Differences in SCV numbers were analyzed by one-way ANOVA with Tukey’s *post hoc* test. ^∗^*P* < 0.05.

To assess the proportion of SCV and WT colonies detectable in axenic culture of *S. aureus*, stationary phase (24 h culture) NCTC 12493, 8532, 8325-4, Newman, and MRSA 252 strains of *S. aureus* were inoculated onto solid media containing gentamicin to select for gentamicin-resistant SCVs [Mueller–Hinton agar containing 2 μg/mL gentamicin (MHA+G)], and antibiotic-free media to enumerate WT colonies (LA) (Figure 1A). SCVs were identified for all strains occurring at frequencies ranging from 1 × 10^–4^ to 2 × 10^–2^ (Figure 1B). Additionally, we observed quantitatively different SCV populations between strains, notably a larger proportion of SCVs were found in Newman compared to 8325-4 [9730 ± 994 vs 4960 ± 876 cfu/mL (*P* = 0.02)]. This difference was found not to be significant when we corrected for multiple comparisons (Figure 1A). Interestingly, there was a tendency for strain 8532 to produce SCVs at a higher frequency (Figure 1A).

**FIGURE 2:**
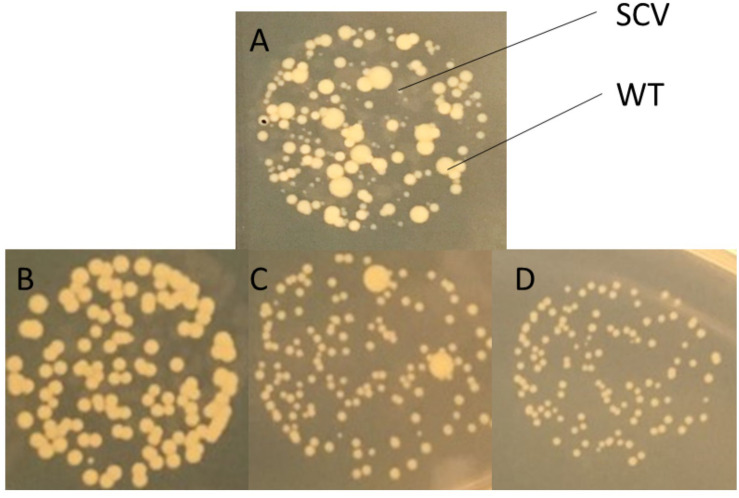
Identification of *S. aureus* SCVs on solid media. **(A)** SCVs were identified by their appearance on Luria-Bertani agar (LA) generally appearing to be <1/10th the size of wild-type colonies. SCVs selected on gentamicin demonstrated either **(B)** full reversion, **(C)** partial reversion, or **(D)** no reversion to wild-type following 24 h growth on non-selective media (LA). Images shown are close-ups of drops on agar demonstrating different colony morphotypes at 24 h.

To examine the dynamics of the SCV population over time, we extended culture of *S. aureus* strains 8325-4 and Newman up to 7 days with the hypothesis that nutrient depletion and acidification of the media would select for SCV formation by favoring slow-growing phenotypes. Newman was examined as it has previously been shown to persist within macrophages and we hypothesized that this ability may be linked to its ability to form SCVs in nutrient deplete and acidic conditions (Jubrail et al., 2016).8325-4 has been used to generate SCVs *in vitro* (von Eiff et al., 1997). We found that while the proportion of gentamicin resistant SCVs did not increase, a second population with an SCV phenotype that was not gentamicin resistant was detectable on antibiotic-free media after 120 h at a frequency approaching that of WT (Figures 1C,D). At 120 h, *S. aureus* colonies showed significant phenotypic variation on solid media (Figure 2A).

In order to examine the stability of the SCV phenotype, Newman strain *S. aureus* was inoculated onto gentamicin containing media (MHA+G). Small colonies that arose were passaged onto gentamicin-free media (LA). Multiple colonies were picked based on morphological characteristics (size and pigmentation). Colonies demonstrated three different behaviors: full reversion to WT, partial reversion, or a stable SCV phenotype (Figure 2). One, termed SCV3, was selected for further work as it demonstrated a stable phenotype over eight passages on antibiotic-free media.

### SCV3 Has a Phenotype Typical of Stable SCVs

**FIGURE 3:**
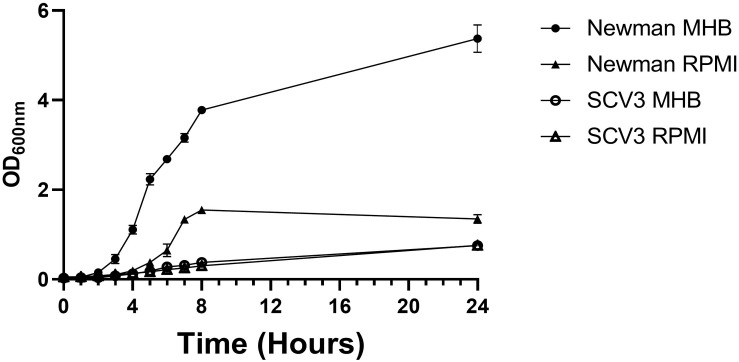
SCV3 shows altered growth kinetics to parent strain Newman *S. aureus*. Newman and SCV3 were inoculated into MHB and RPMI-1640 and incubated on a shaking incubator at 37°C for 24 h. Growth was measured by optical density at 600 nm. Data points represent the mean of three independent experiments. Error bars demonstrate standard deviation of the mean.

To characterize SCV3, we first examined its growth kinetics compared to WT Newman strain *S. aureus* in MHB and RPMI 1640 (Figure 3). RPMI 1640 has a composition designed to mimic human tissue fluid but also supports the growth of *S. aureus.* As expected, SCV3 showed a markedly reduced doubling time (124 vs 44 min) over 24 h compared to WT Newman, with SCV3 only reaching an optical density in MHB of 0.8 ± 0.005 after 24 h compared to 5.4 ± 0.2 for WT. Second, we assessed the antibiotic susceptibility of SCV3 and WT Newman by microbroth dilution. Growth was measured by optical density at 600 nm on a plate reader at 24 h for WT Newman and 48 h for SCV3. Only the MIC for gentamicin was increased beyond one dilution difference compared with the parent strain (Table 1) (Clinical and Laboratory Standards Institute, 2012).

**TABLE 1:** SCV3 shows resistance to Gentamicin but retains sensitivity to other antibiotics.

	MIC (μg/mL)	
Antibiotic	Newman	SCV3
Gentamicin	0.63	10
Flucloxacillin	0.33 (0.11)	0.20
Erythromycin	1.25	0.63
Ofloxacin	0.39	0.20
Rifampicin	0.005	0.01

*MICs were determined at 24 h for Newman and 48 h for SCV3. MICs of SCV3 and wild-type were within one dilution, except for gentamicin that was used to select SCV3. Values are derived from three biological replicates each of three technical replicates. Where there was discrepancy between biological replicates, the value shown is the mean with the standard deviation in parentheses.*

### Intracellular Survival Within Macrophages Does Not Select for SCVs

**FIGURE 4:**
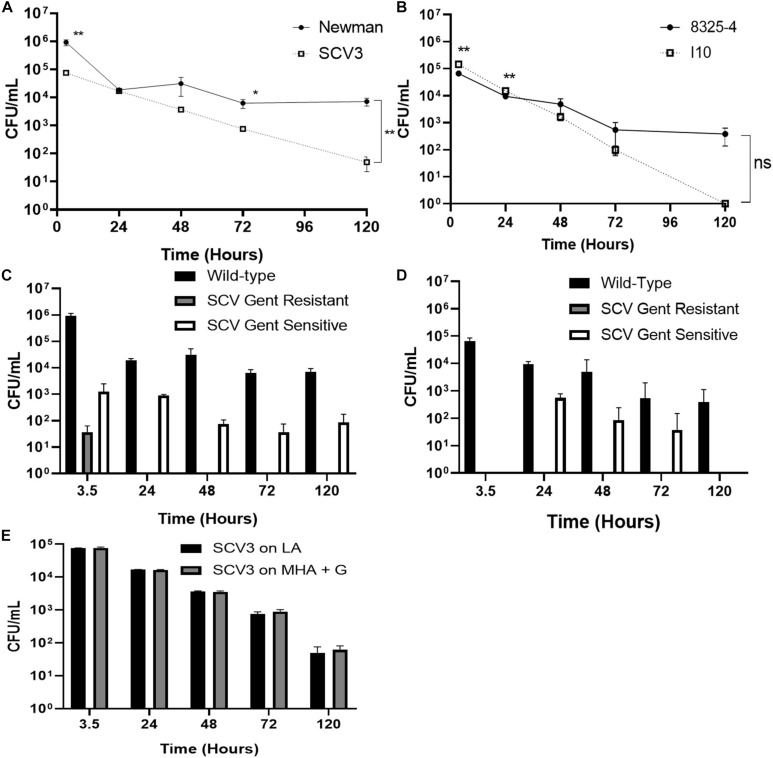
Intracellular survival within macrophages does not select for SCVs. Differentiated THP-1 macrophages were infected with different strains of *S. aureus* at an MOI of 1. After 3 h, extracellular bacteria were removed by washing and by treatment with lysostaphin. Intracellular bacteria were plated onto media with (MHA+G) or without (LA) gentamicin. Colony forming units of **(A)** Newman and corresponding SCV3 and **(B)** 8325-4 and its derivative SCV strain I10 were measured over 5 days. Intracellular SCV3 numbers were significantly lower than that of Newman at day 5. At 5 days, no colonies of I10 were recovered on any media. Enumeration of different colony phenotypes recovered from macrophages after 5 days infection with **(C)** Newman and **(D)** 8325-4. No SCVs were recovered on MHA+G for 8325-4 and none beyond 3.5 h for Newman. A small population of SCVs was observed in the absence of gentamicin. **(E)** SCV3 did not revert to wild-type colony morphology during intracellular infection. There was no significant difference between numbers of colonies on either media type. Data points represent the mean of three separate experiments each with three technical replicates ± SEM. Means were compared by independent *t*-test. *P < 0.05, **P < 0.01.

To test the hypothesis that SCVs represent an adapted intracellular phenotype within professional phagocytes, we examined selection for, and persistence of, SCVs within THP-1 derived macrophages. PMA stimulated THP-1 derived macrophages were infected with WT, Newman and 8325-4 *S. aureus*, and their corresponding SCVs, SCV3, and I10 (Figures 4A,B). Intracellular killing of Newman was incomplete up to 5 days (Figure 4A), with ∼2 log reduction in viable intracellular bacteria over 120 h. 8325-4 showed considerable variability in survival at 120 h (Figure 4B). The corresponding SCVs were killed more effectively by macrophages. By day 5 intracellular numbers of SCV3 and I10 were markedly less than those of WT Newman and 8325-4, respectively (Figures 4A,B).

Colonies cultured from intracellular bacteria were examined for SCV phenotype on both antibiotic-free (LA) and gentamicin-containing (MHA+G) media. Although a small population of gentamicin-resistant SCVs was initially identified at 3.5 h with Newman, this was not detectable at later time points (Figure 4C), or at all in 8325-4 (Figure 4D). There was a consistent increase in the proportion of gentamicin-sensitive SCVs at 24 h (Figures 4C,D) coinciding with the highest rate of intracellular killing. However, the proportion of these colonies fell by the end of the experiment.

To determine whether the intracellular environment might exert selection pressure to cause SCV3 to revert to WT, we measured changes in colony morphology over time in SCV3 isolated from the intracellular compartment. We found the SCV phenotype and gentamicin resistance was maintained with no significant difference in CFUs between colonies isolated on gentamicin-containing and antibiotic-free media (Figure 4E).

## Discussion

Small-colony variant formation has been proposed as a mechanism by which *S. aureus* evades the host immune response, establishes chronic infection, and remains refractory to treatment (Proctor et al., 2006). Recent findings suggest that SCV formation may be a constitutive part of *S. aureus* growth, adopted as a bet-hedging strategy against changing environmental conditions (Edwards, 2012).

In keeping with this hypothesis, we have confirmed previous findings that SCVs arise at low frequencies during axenic culture of all strains of *S. aureus* tested, in the absence of obvious selection pressure. These observations mirror those for other *S. aureus* strains following exposure to gentamicin (Edwards, 2012; Vestergaard et al., 2016). Several mutations conferring reduced aminoglycoside susceptibility have been described in both clinically isolated and laboratory-constructed SCVs (Schaaff et al., 2003). Gentamicin resistance provides a useful selection tool for SCVs arising from disruption of the electron transport chain (Schaaff et al., 2003; von Eiff et al., 2006). Loss of membrane potential reduces susceptibility to the heavily charged aminoglycoside molecule. Gentamicin selection is particularly useful as it inhibits the growth of WT *S. aureus* but allows for the continued growth of SCVs in mixed culture. However, not all SCVs arising spontaneously will be gentamicin resistant, particularly short-lived unstable SCVs. To address this limitation and, in contrast to previous studies, we have enumerated SCVs both on antibiotic-free media (LA) and on gentamicin-containing media. We observed that in extended culture, a population of gentamicin-sensitive SCVs arose to magnitudes comparable to WT bacteria. This represents the first description of mixed SCV populations, distinguishable by their gentamicin sensitivity, arising spontaneously during prolonged growth. Thus, these data support the prevailing hypothesis that SCVs represent a compound phenotype of revertant and stable morphotypes arising from multiple mechanisms rather than a single regulatory or mutational switch (Kahl et al., 2016).

Small-colony variant identification on antibiotic-free media has a significant limitation in that slow-growing colonies will not be identified by the surface viable count method, in populations of *S. aureus* where the majority of bacteria give rise to large colonies. Large colonies will overwhelm SCVs present at low frequencies. Automated plate readers and single cell imaging have provided a useful way around this problem (Vulin et al., 2018). Vulin et al. (2018) were able to demonstrate SCVs arising due to delayed initiation of growth, rather than reduced growth rate using this technique. In the present study, the viable count method was able to identify SCVs on non-selective media when their number closely resembled that of WT, allowing for enumeration at the same dilution.

We find statistically significant differences in the number of SCVs produced by different SCV strains, notably NCTC 8532 produced significantly more SCVs than MRSA 252, Newman, and 8325-4. The biological significance of this remains unclear. It would be interesting to investigate whether these differences are associated with an increased propensity to establish chronic infection.

While SCV formation is promoted by conditions mimicking the intracellular environment (Painter et al., 2015; Leimer et al., 2016; Vulin et al., 2018), and SCVs appear to have a survival advantage within non-professional phagocytes (von Eiff et al., 1997; Tuchscherr et al., 2011; Kalinka et al., 2014; Leimer et al., 2016; Rollin et al., 2017), there have been limited data available from phagocytic cells. Three studies have examined intracellular killing of SCVs within THP-1 macrophage-like cells over 72 h in response to antibiotics (Barcia-Macay et al., 2006; Nguyen et al., 2009; Sandberg et al., 2011; Painter et al., 2015). The behavior of THP-1 macrophages varies greatly according to the extent of differentiation (Daigneault et al., 2010). Thus, it is difficult to draw conclusions from these experiments on macrophage killing of SCVs. Indeed, the aim of these studies was to establish a stable intracellular population that could be exposed to antibiotics; in their control experiments extracellular bacteria were allowed to persist and maintain an intracellular population. Intracellular survival of WT *S. aureus* within macrophages and neutrophils has been demonstrated by several studies (Kubica et al., 2008; Painter et al., 2015; Jubrail et al., 2016) and has been hypothesized to represent a vehicle for dissemination of infection (Thwaites and Gant, 2011). Lehar et al. (2015) showed that mice infected with intracellular *S. aureus* still produced abscesses despite concurrent treatment with antibiotics. Data examining SCV interactions with phagocytes are far more limited. Painter et al. demonstrated that SCVs were more likely to survive exposure to human neutrophils than WT, despite similar levels of internalization over 3 h (von Eiff et al., 2006). By contrast Tuchscherr et al. (2011) found elimination of *S. aureus* from macrophage with no evidence of SCV formation. Here, we have shown for the first time that WT *S. aureus* has a survival advantage over SCVs in THP-1 macrophages during prolonged infection. We used a method based on that of Jubrail et al. (2016) who were able to demonstrate intracellular survival of WT *S. aureus* in both THP-1 and monocyte derived macrophages. Interestingly the rate of killing is higher for Newman than SCV 3 (Figure 4A) implying that early killing may favor SCVs consistent with previous findings (Painter et al., 2015). Jubrail et al. (2016) demonstrated a failure of lysosomal acidification in THP-1 macrophages infected with *S. aureus*, while Staphylococcus *epidermidis* SCVs have recently been shown to colocalize at higher rates within lysosomes than their WT counterparts (Magryś et al., 2018); it is possible that SCVs are unable to impair lysosomal acidification to the same extent as their WT counterparts. Additionally, Jubrail et al. (2016) demonstrated repeated cycles of macrophage lysis and re-uptake which may be impaired in SCVs.

One characteristic feature of many studies examining intracellular survival is the use of high MOI (c. 100) to achieve an intracellular infection, which may not be representative of *in vivo* host–pathogen interactions. Here we have used a low MOI of 1 to achieve intracellular persistence with the WT strain Newman, hoping to prevent saturation of intracellular killing mechanisms simply by weight of numbers.

Our study has several important limitations: We have only examined the elimination of two SCV strains, one arising from gentamicin selection (SCV3), a common feature of clinically isolated SCVs, and one laboratory constructed mutant (I10). It is difficult to generalize these findings to all SCVs. Certainly intracellular survival of WT *S. aureus* is strain specific (Strobel et al., 2016), this is not well described for SCVs. Additionally, the behavior of THP-1 macrophages *in vitro* may not be similar enough to tissue macrophages *in vivo*, despite previous observations that THP-1 macrophages and monocyte-derived macrophages responded similarly to WT *S. aureus* (Jubrail et al., 2016). Nevertheless none of these limitations undermine the fundamental observation that THP-1 macrophages eliminated both gentamicin-selected and laboratory-constructed SCVs more effectively than their WT counterparts (Figures 4A,B) and infection with WT *S. aureus* did not give rise to enrichment of the SCV population (Figures 4C,D). Future studies are required to examine the interaction of SCVs with macrophages *in vivo*.

## Conclusion

Our data support previous findings that SCV formation appears to be a constitutive part of *S. aureus* growth and that this gives rise to mixed populations of gentamicin-sensitive and gentamicin-resistant SCVs. While this process may play an important role in tolerance to aminoglycosides, and establishing chronic infection, we find no evidence for spontaneously arising SCVs having a survival advantage over their WT counterparts within THP-1 macrophages.

## Data Availability Statement

All datasets generated/analyzed for this study are included in the article/supplementary material.

## Author Contributions

SS, ML, and SW conceived and designed the study. Experiments were performed by SS and DC. SS and DC analyzed the data. SS wrote the first draft of the manuscript. ML and SW reviewed the manuscript. All authors read and approved the final manuscript.

## Conflict of Interest

The authors declare that the research was conducted in the absence of any commercial or financial relationships that could be construed as a potential conflict of interest.
